# Supplementary material for: (R)-Salbutamol Improves Imiquimod-Induced Psoriasis-Like Skin Dermatitis by Regulating the Th17/Tregs Balance and Glycerophospholipid Metabolism
Source: Cells. 2020 Feb 24;9(2):511. doi: 10.3390/cells9020511 (PMC7072797; doi:10.3390/cells9020511)
Supplement: Supplementary file 1 [file cells-09-00511-s001.pdf]

SUPPLEMENTARY

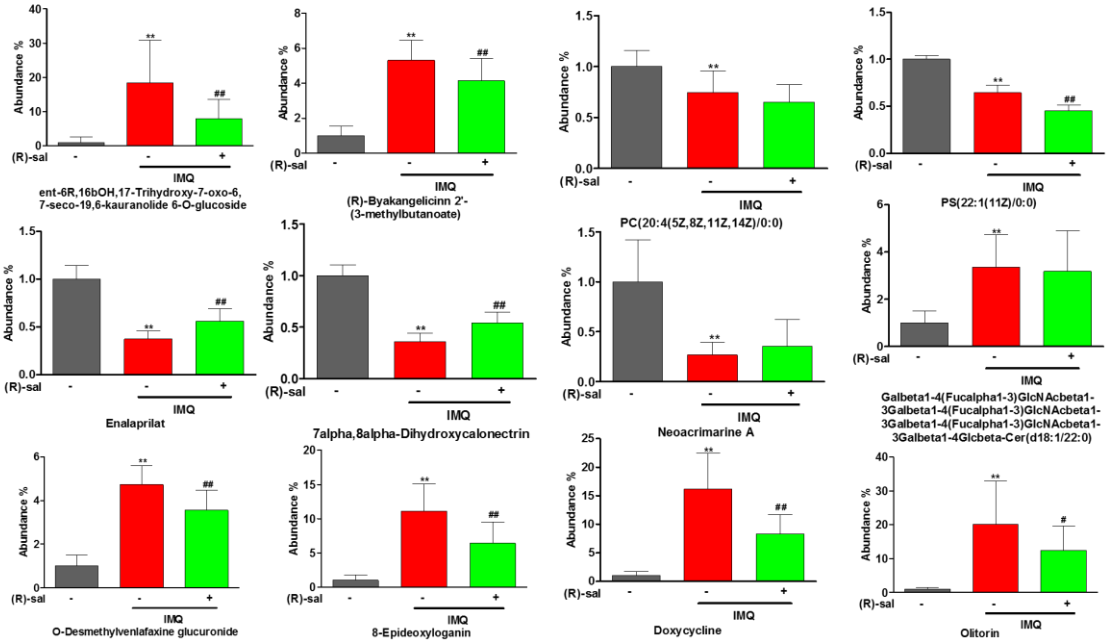

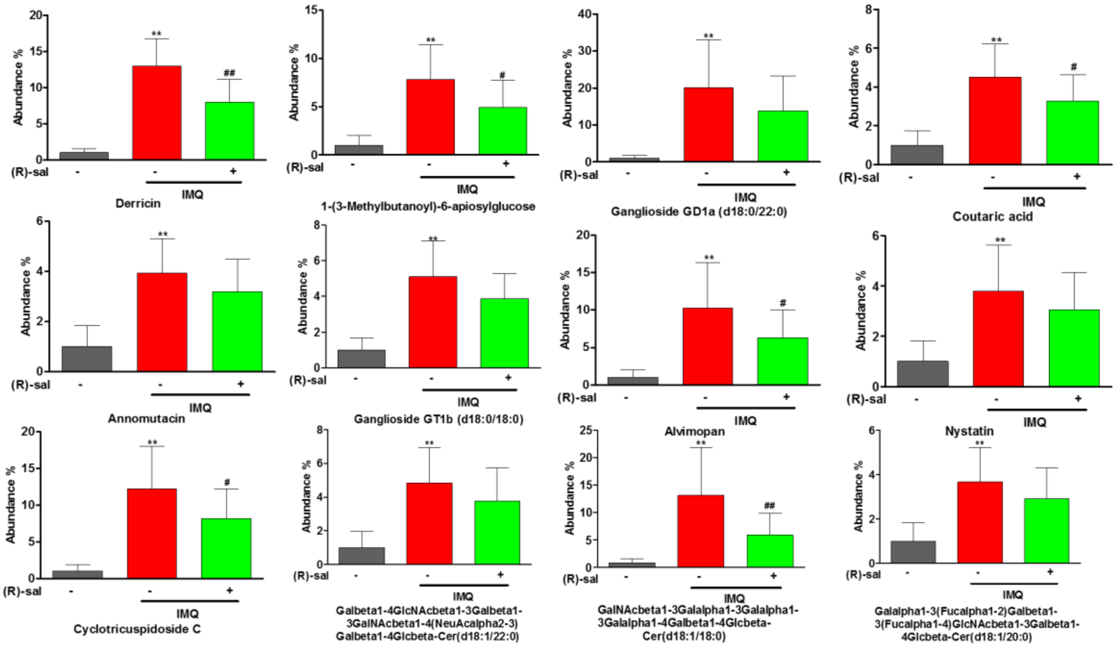

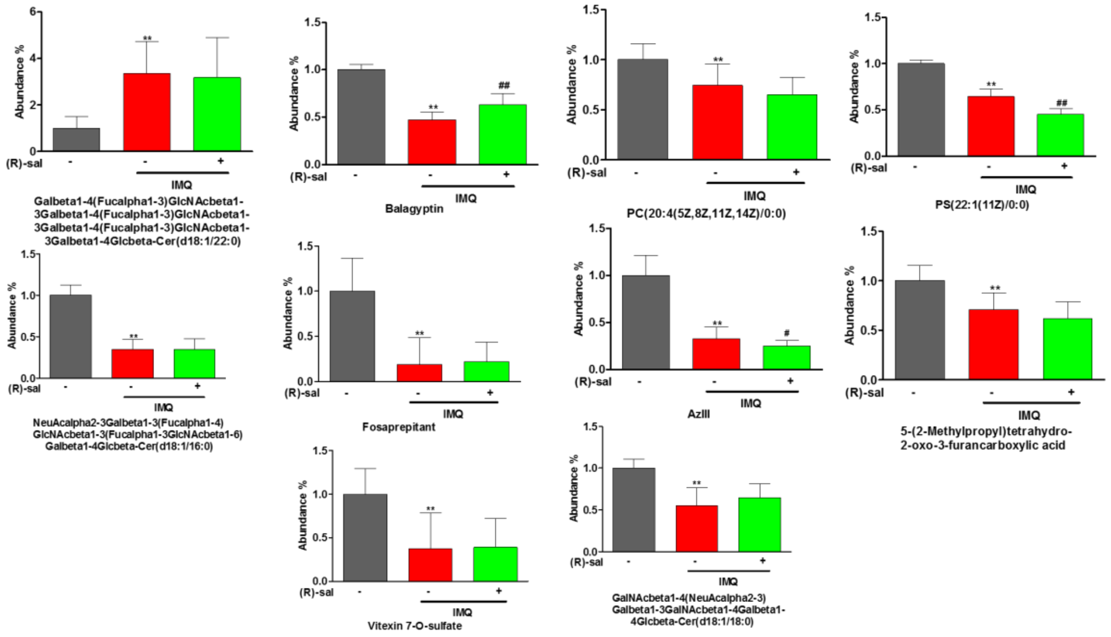

SUPPLEMENTARY Figure 1 *Potential biomarkers identified by UHPLC-TIMS-TOF MS/MS*

SUPPLEMENTARY TABLE 1. *Psoriasis area and severity index (PASI) score*

	Erythema	*Scaling*	*Skin thickness*
none	*0*	*0*	*0*
slight	*1*	*1*	*1*
moderate	*2*	*2*	*2*
marked	*3*	*3*	*3*
very marked	*4*	*4*	*4*

SUPPLEMENTARY TABLE 2. *Pathological score by Baker score system*

Keratinocyte	*Epidermis*	*Dermis*
Munro abscess 2.0	*Acanthosis 1.0*	*Angiotelectasis 0.5*
Hyperkeratosis 0.5	*Lengthening and clubbing of rete ridges slight0.5, moderate1,marked1.5*	*Inflammatory cells infiltration slight0.5, moderate1,marked1.5*
Parakeratosis 1.0	*Lack of granular layer 1.0*	*Papillary congestion 0.5*

SUPPLEMENTARY TABLE 3. *QC samples-based investigation of the analytical methods (accuracy (<10%), repeatability (<15%) and stability (<20%)). A total of six ions in positive ion mode and six ions in negative ion mode from the extracted ion chromatographic peaks were selected for method validation. The RSDs of retention time for injection precision, repeatability and system stability were estimated.*

Scan polarity	Ion pair	Precision				Repeatability				System Stability			
		RT (min)		Area		RT (min)		Area		RT (min)		Area	
		X	RSD%	X	RSD%	X	RSD%	X	RSD%	X	RSD%	X	RSD%
ESI (+)	0.40-203.0305	0.40	0.00	355379.90	5.81	0.40	0.00	431603.20	10.20	0.40	0.00	463265.70	12.13
	2.50-406.6684	2.50	0.00	741936.50	6.87	2.50	0.00	744486.10	8.95	2.44	2.24	1268432.00	17.11
	4.00-437.8683	4.00	0.00	277869.40	3.67	4.00	0.00	261172.00	3.50	4.00	0.00	190092.90	17.47
	6.00-550.9698	6.00	0.00	261866.10	3.32	6.00	0.00	94776.65	1.05	6.00	0.00	80856.93	15.05
	12.35-496.2848	12.35	0.44	2625301.00	2.41	12.34	0.44	2509685.00	12.82	12.30	0.00	2617959.00	1.20
	13.60-524.3127	13.6	0.00	1279326.00	3.21	13.60	0.00	1167062.00	11.63	13.60	0.00	1237283.00	4.26
ESI (-)	0.40-215.0183	0.40	0.00	160369.30	6.29	0.10	0.00	157992.30	4.26	0.40	0.00	167166.60	12.24
	2.10-203.0688	2.10	0.00	413523.90	5.43	2.10	0.00	412092.60	4.19	2.18	2.05	421801.00	7.50
	4.20-348.1693	4.20	0.00	469627.80	7.57	4.20	0.00	522278.30	8.67	4.28	1.04	594834.70	6.61
	11.50-538.2785	11.50	0.00	221996.50	7.64	11.50	0.00	201156.70	3.31	11.50	0.00	219021.40	6.71
	13.30-568.3236	13.30	0.00	267063.60	5.37	13.30	0.00	177775.60	10.48	13.30	0.00	216427.60	7.94
	15.80-281.2295	15.80	0.00	358268.40	8.00	15.80	0.00	238267.80	5.90	15.80	0.00	349417.80	12.89

SUPPLEMENTARY TABLE 4. *Potential biomarkers of anti-psoriasis in (R)-salbutamol-treated IMQ-induced mouse psoriasis obtained from UHPLC/ESI-TIMS TOF-MS/MS*

NO.	*RT (min)a*	*m/z*	*Mode*	*Biomarker identification*	*Formula*	*Trend b*	*Trend c*	*Related pathway*
1	*2.47*	*279.0798*	*d N*	*N-Despyridinyl rosiglitazone*	*C13H16N2O3S*	↑ **	↓ ##	
2	*4.44*	*342.2162*	*N*	*Dibucaine*	*C20H29N3O2*	↑ **	↓ ##	
3	*4.03*	*314.1871*	*N*	*Saxagliptin*	*C18H25N3O2*	↑ **	↓ #	
4	*1.55*	*463.1633*	*N*	*PSF-A*	*C22H26O8*	↑ **	↓ ##	
5	*4.84*	*755.3318*	*N*	*Olitorin*	*C35H52O14*	↑ **	↓ #	
6	*2.65*	*573.2501*	*N*	*ent-6R,16bOH,17-Trihydroxy-7-oxo-6,7-seco-19,6-kauranolide 6-O-glucoside*	*C26H40O11*	↑ **	↓ ##	
7	*4.21*	*835.3256*	*N*	*(R)-Byakangelicinn 2′-(3-methylbutanoate)*	*C22H26O8*	↑ **	↓ #	
8	*4.13*	*590.2428*	*N*	*Petasinoside*	*C28H37NO9*	↑ **	↓ ##	
9	*4.06*	*881.3793*	*N*	*O-Desmethylvenlafaxine glucuronide*	*C20H29NO8*	↑ **	↓ ##	
10	*0.67*	*373.1471*	*N*	*8-Epideoxyloganin*	*C17H26O9*	↑ **	↓ ##	
11	*0.85*	*461.1572*	*N*	*Doxycycline*	*C22H26N2O9*	↑ **	↓ ##	
12	*4.84*	*755.8333*	*N*	*11Z-Heptacosene*	*C27H54*	↑ **	↓ #	
13	*4.87*	*703.3165*	*N*	*Derricin*	*C21H22O3*	↑ **	↓ ##	
14	*1.10*	*397.1718*	*P*	*1-(3-Methylbutanoyl)-6-apiosylglucose*	*C16H28O11*	↑ **	↓ #	
15	*4.16*	*639.688*	*P*	*Ganglioside GD1a (d18:0/22:0)*	*C89H159N3O39*	↑ **	-	
16	*4.27*	*350.2070*	*P*	*Coutaric acid*	*C18H27N3O4*	↑ **	↓ #	
17	*4.75*	*688.5180*	*P*	*Annomutacin*	*C37H68O7*	↑ **	-	
18	*5.16*	*718.0301*	*P*	*Ganglioside GT1b (d18:0/18:0)*	*C96H168N4O47*	-	-	
19	*3.32*	*442.2658*	*P*	*Alvimopan*	*C25H32N2O4*	↑ **	↓ #	
20	*3.84*	*474.7505*	*P*	*Nystatin*	*C47H75NO17*	↑ **	-	

21	*4.08*	*442.2324*	*P*	*Cyclotricuspidoside C*	*C43H72O17*	↑ **	↓ #	
22	*5.30*	*664.0288*	*P*	*Galbeta1-4GlcNAcbeta1-3Galbeta1-3GalNAcbeta1-4(NeuAcalpha2-3)Galbeta1-4Glcbeta-Cer(d18:1/22:0)*	*C91H162N4O41*	↑ **	-	
23	*4.07*	*534.6280*	*P*	*GalNAcbeta1-3Galalpha1-3Galalpha1-3Galalpha1-4Galbeta1-4Glcbeta-Cer(d18:1/18:0)*	*C74H134N2O33*	↑ **	↓ ##	*Sphingolipid metabolism*
24	*4.79*	*587.3268*	*P*	*Galalpha1-3(Fucalpha1-2)Galbeta1-3(Fucalpha1-4)GlcNAcbeta1-3Galbeta1-4Glcbeta-Cer(d18:1/20:0)*	*C82H148N2O36*	↑ **	-	*Sphingolipid metabolism*
25	*5.04*	*834.7642*	*P*	*Galbeta1-4(Fucalpha1-3)GlcNAcbeta1-3Galbeta1-4(Fucalpha1-3)GlcNAcbeta1-3Galbeta1-4(Fucalpha1-3)GlcNAcbeta1-3Galbeta1-4Glcbeta-Cer(d18:1/22:0)*	*C112H198N4O55*	↑ **	-	*Sphingolipid metabolism*
26	*3.40*	*481.2887*	*P*	*PI(20:4(5Z,8Z,11Z,14Z)/22:2(13Z,16Z))*	*C51H87O13P*	↑ **	↓ #	
27	*11.73*	*544.3378*	*P*	*PC(20:4(5Z,8Z,11Z,14Z)/0:0)*	*C28H50NO7P*	↓ **	-	*Glycerophospholipid metabolism*
28	*11.91*	*544.3380*	*P*	*PS(22:1(11Z)/0:0)*	*C28H54NO9P*	↓ **	↑ ##	*Glycerophospholipid metabolism*
29	*11.88*	*568.3379*	*P*	*PC(20:3(8Z,11Z,14Z)/0:0)*	*C28H52NO7P*	↓ **	↑ ##	*Glycerophospholipid metabolism*
30	*5.34*	*406.2048*	*P*	*Balagyptin*	*C39H64O16*	↓ **	↑ ##	
31	*0.47*	*204.1231*	*P*	*5-(2-Methylpropyl)tetrahydro-2-oxo-3-furancarboxylic acid*	*C9H14O4*	↓ **	-	
32	*4.54*	*553.2622*	*P*	*AzIII*	*C54H82O22*	↓ **	↓ #	
33	*5.67*	*656.1554*	*P*	*Fosaprepitant*	*C23H22F7N4O6P*	↓ **	-	

34	*4.74*	*679.3537*	*P*	*NeuAcalpha2-3Galbeta1-3(Fucalpha1-4)GlcNAcbeta1-3(Fucalpha1-3GlcNAcbeta1-6)Galbeta1-4Glcbeta-Cer(d18:1/16:0)*	*C91H160N4O44*	↓ **	-	*Sphingolipid metabolism*
35	*5.98*	*551.0294*	*P*	*Vitexin 7-O-sulfate*	*C21H20O13S*	↓ **	-	
36	*6.27*	*886.4762*	*P*	*GalNAcbeta1-4(NeuAcalpha2-3)Galbeta1-3GalNAcbeta1-4Galbeta1-4Glcbeta-Cer(d18:1/18:0)*	*C81H144N4O36*	↓ **	-	
37	*3.73*	*755.3207*	*N*	*Enalaprilat*	*C18H24N2O5*	↓ **	↑ ##	
38	*5.35*	*809.3298*	*N*	*7alpha,8alpha-Dihydroxycalonectrin*	*C19H26O8*	↓ **	↑ ##	
39	*4.33*	*26.2965*	*N*	*Neoacrimarine A*	*C40H43NO9*	↓ **	-	
